# Predicting relapse after antidepressant withdrawal – a systematic review

**DOI:** 10.1017/S0033291716002580

**Published:** 2016-10-27

**Authors:** I. M. Berwian, H. Walter, E. Seifritz, Q. J. M. Huys

**Affiliations:** 1 Department of Psychiatry, Psychotherapy and Psychosomatics, Hospital of Psychiatry, University of Zurich, Lenggstrasse 31, 8032 Zürich, Switzerland; 2 Translational Neuromodeling Unit, Institute for Biomedical Engineering, University of Zurich and ETH Zurich, Wilfriedstrasse 6, 8032 Zürich, Switzerland; 3 Mind and Brain, Campus Charité Mitte, Charité Universitätsmedizin Berlin, Charitéplatz 1, 10117 Berlin, Germany

**Keywords:** Antidepressant discontinuation, major depressive disorder, prediction, relapse

## Abstract

A substantial proportion of the burden of depression arises from its recurrent nature. The risk of relapse after antidepressant medication (ADM) discontinuation is high but not uniform. Predictors of individual relapse risk after antidepressant discontinuation could help to guide treatment and mitigate the long-term course of depression. We conducted a systematic literature search in PubMed to identify relapse predictors using the search terms ‘(depress* OR MDD*) AND (relapse* OR recurren*) AND (predict* OR risk) AND (discontinu* OR withdraw* OR maintenance OR maintain or continu*) AND (antidepress* OR medication OR drug)’ for published studies until November 2014. Studies investigating predictors of relapse in patients aged between 18 and 65 years with a main diagnosis of major depressive disorder (MDD), who remitted from a depressive episode while treated with ADM and were followed up for at least 6 months to assess relapse after part of the sample discontinued their ADM, were included in the review. Although relevant information is present in many studies, only 13 studies based on nine separate samples investigated predictors for relapse after ADM discontinuation. There are multiple promising predictors, including markers of true treatment response and the number of prior episodes. However, the existing evidence is weak and there are no established, validated markers of individual relapse risk after antidepressant cessation. There is little evidence to guide discontinuation decisions in an individualized manner beyond overall recurrence risk. Thus, there is a pressing need to investigate neurobiological markers of individual relapse risk, focusing on treatment discontinuation.

## Introduction

Depression is a major health issue (Whiteford *et al.* 2013), and a substantial proportion of its burden arises through relapses and chronic courses: more than half of those with a first episode of depression will go on to have a second, and the majority of them will have further episodes (American Psychiatric Association, 2000). Preventing relapses of depression is hence a critical component of treatment. Antidepressant medications (ADM) have proven utility not only in treating acute episodes of depression, but also in reducing the risk of relapse (Geddes *et al.* 2003; Kaymaz *et al.* 2008; Glue *et al.* 2010; Sim *et al.* 2015). However, they are no panacea: not all patients respond to ADMs, particularly not first-line ones (e.g. Rush *et al.* 2006); many experience side-effects; thus, discontinue treatment rapidly (Olfson *et al.* 2006) or adhere partially (Hunot *et al.* 2007); some relapse despite ADM maintenance treatment while others do not relapse despite discontinuation. Of note, there are also suggestions that antidepressant treatment itself might contribute to relapses after discontinuation through perturbational effects on neuromodulatory systems (Andrews *et al.* 2011).

A substantial effort is now underway to improve acute treatment response rates by improving how agents are targeted to individual patients (e.g. ISPOT-D; CAN-BIND; IMAGEMEND), with some promising initial results (e.g. Palmer, 2015). However, the other side of the treatment, the decision about whether to move to (possibly long-term) maintenance therapy or to discontinue, has attracted far less research focused on making individualized predictions. Given the importance of the initial episodes of depression in setting up the long-term course of the disorder (e.g. Kendler *et al.* 2000; Monroe & Harkness, 2005, 2011), this is a very pressing research lacuna.

Guidelines [National Collaborating Centre for Mental Health (UK), 2010; Bauer *et al.* 2013] currently recommend a continuation of treatment after an initial response for 4 to 9 months and maintenance treatment of 2 years or longer in the case of recurrent major depressive disorder (MDD). As such, the main indices that are used to inform the termination of maintenance treatment are the treatment duration and the number of prior episodes or chronicity. Though these recommendations are well motivated by the natural course of depressive episodes (Frank *et al.* 1991; Kessler *et al.* 2003) and the protective effect of antidepressants, to our knowledge there are no systematic examinations of what predictors relate to discontinuation above and beyond overall relapse risk. We hence here aim to provide a systematic review of the existing evidence.

Overall relapse risk and relapse risk after discontinuation may be only partially related: some patients will relapse independently of whether they are prescribed any medication. Notably, although such high-risk patients are typically prime targets for treatment, they would derive as little advantage from the medication as those who will not relapse either way.

**Fig. 1.**
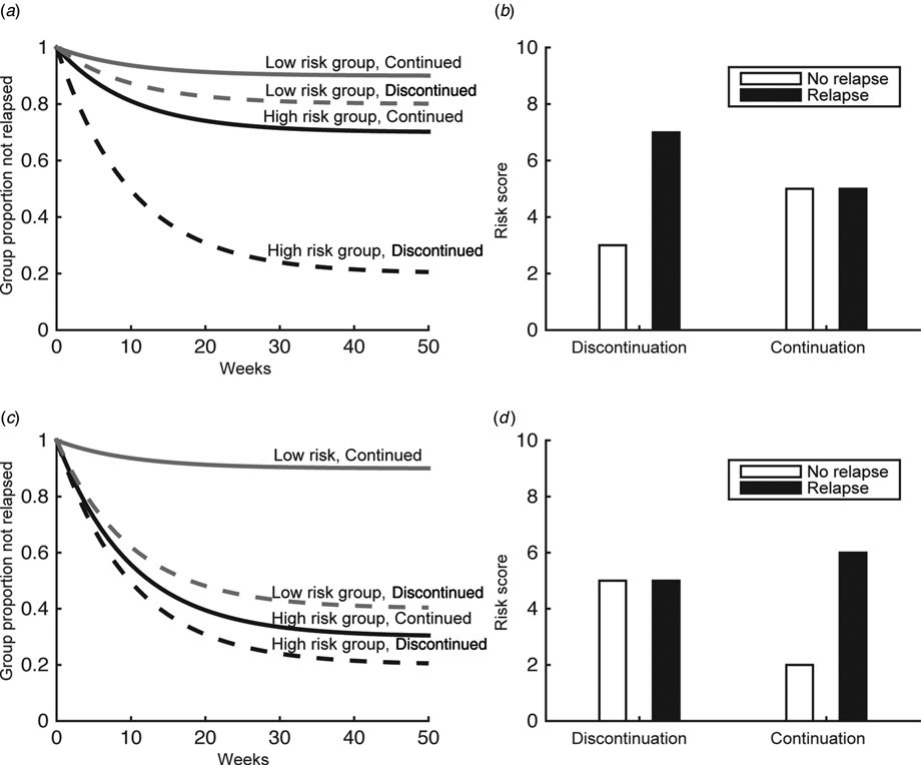
Relapse risk after placebo-controlled randomized antidepressant treatment discontinuation. Some risk factors may identify patients who benefit from antidepressant medication due to effects driven by discontinuation (*a*), or continuation arms (*c*). In the former case, the risk score should differ between relapsers and non-relapsers in the discontinuation arm (*b*), while in the latter case it should differ in the continuation arm (*d*). Other risk factors may identify mixed effects.

Hence, it is critical to establish predictors of the relapse risk for individual patients. In terms of clinical guidance, this raises two related questions. The first asks what the individual's relapse risk would be after discontinuation. Patients who have a very low risk of relapse after discontinuation have less scope for benefiting from further treatment (grey lines, Fig. 1*a*) than those with high risk (black lines, Fig. 1*a*). This requires putative predictive variables to differentiate between relapsers and non-relapsers specifically after discontinuation. To establish, second and more generally, what the benefit of continued treatment in a particular patient is, the relapse risk after discontinuation has to be compared with the relapse risk under continued medication and thus requires examination of interactions, comparing the difference between relapsers and non-relapsers in continuation and discontinuation arms (Fig. 1*b*). We examined both of these, i.e. searched for studies that either reported a difference between relapsers and non-relapsers specifically after discontinuation; or an interaction of treatment (continuation/discontinuation) with a putative predictor in differentiating relapsers and non-relapsers. That is, we were particularly interested in whether there was a treatment × predictor interaction between relapsers and non-relapsers. Note, however, that an interaction can be driven by the continuation arm alone, too, and thereby be relatively uninformative about discontinuation (Fig. 1*c, d*).

## Method

We conducted a systematic literature search in PubMed to identify relapse predictors using the search terms ‘(depress* OR MDD*) AND (relapse* OR recurren*) AND (predict* OR risk) AND (discontinu* OR withdraw* OR maintenance OR maintain or continu*) AND (antidepress* OR medication OR drug)’ for published studies until November 2014. The search resulted in 899 retrieved studies.

Inclusion criteria were:

(*a*) Patient population aged between 18 and 65 years;
(*b*) Main diagnosis of MDD;
(*c*) Remitted from a depressive episode while treated with ADM;
(*d*) Follow-up of at least 6 months to assess relapse;
(*e*) Part of the sample discontinued the ADM (discontinuation could either be part of a randomized controlled design, whereby part of the sample received placebo after randomization starting at a predefined point in time or based on the decision of the patient and treating physician as part of a naturalistic design);
(*f*) Original research paper;
(*g*) Reported relapse predictors either in the discontinuation group alone; or reported interaction of treatment × putative predictor in predicting relapse.

Exclusion criteria were:

(*a*) Anonymous data derived from health systems prescription records;
(*b*) Confounds with psychotherapy.

I.M.B. and Q.J.M.H. first screened all titles. Abstracts of all titles judged potentially relevant by either author were then judged on inclusion criteria *a*–*f* and exclusion criteria. The resulting 61 papers were then retrieved in full text and inclusion and exclusion criteria examined by I.M.B. (including, in addition, criterion *g*) and unclear cases discussed jointly. Authors of individual studies were not contacted.

Of note, the natural course of depressive episodes suggests that re-emergence of symptoms within 6–9 months might be due to relapses into the index episode while thereafter they indicate a new episode (Frank *et al.* 1991). We will here refer to both as relapses as there are insufficient data to distinguish relapses from recurrences for the present purpose.

## Results

**Fig. 2.**
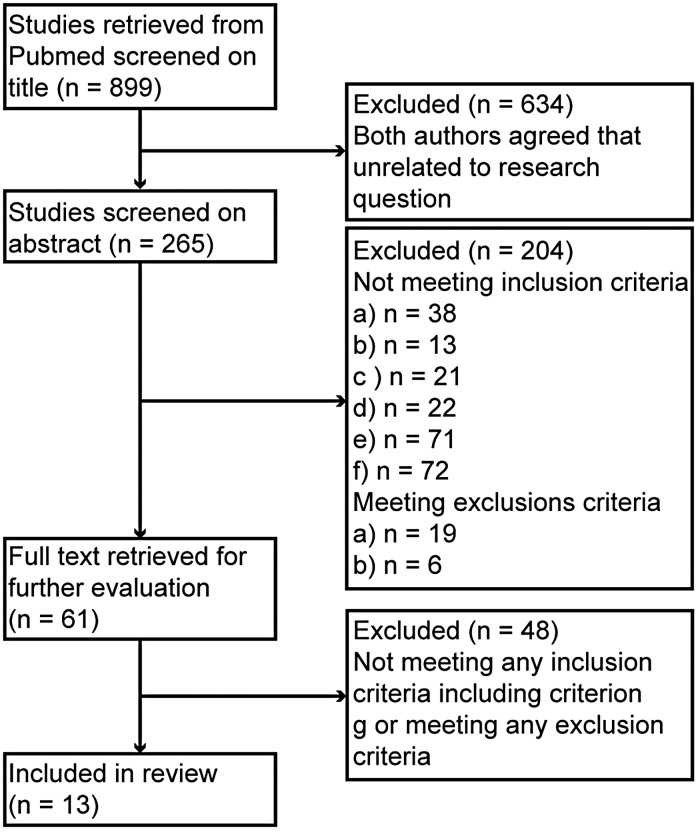
Consolidated Standards of Reporting Trials (CONSORT) diagram depicting number of studies excluded at each step. Exclusion and inclusion criteria are not mutually exclusive. Not all criteria met by each study might be listed in the second box on the right-hand side, since it was not always possible to determine all criteria from the abstract.

Overall, 899 studies were retrieved. Cohen's *κ* for inter-rater agreement for abstracts was 0.75. A total of 61 potentially relevant papers (Fig. 2) were identified. After reading the full-text versions of all these, 13 studies based on nine separate datasets (Table 1) were identified as suitable and included. No study used neuroimaging predictors. Predictors in the categories demographics, disease course, depression subtype and co-morbidity were investigated. An overview of the investigated predictors is shown in Tables 2 and 3. No naturalistic study was identified. All patients who discontinued received placebo treatment. Henceforth, treatment × characteristic interactions always refer to interactions in the prediction of relapse unless otherwise specified.

**Table 1.** Description of datasets

Original datasets	Studies investigated predictors	Diagnostic criteria	Pre-randomization period, weeks	Number of patients per group	Criteria for relapse	Length of follow-up, weeks	Medication
McGrath *et al.* (2006)	McGrath *et al.* (2006), Yang *et al.* (2009), Iovieno *et al.* (2011), Trinh *et al.* (2011)	DSM-IV	12	Fluoxetine = 131, placebo = 131	Two consecutive weeks of ratings of less than ‘much improved’ on the CGI improvement scale compared with ratings at entry into the study	52	Fluoxetine
Hochstrasser *et al.* (2001)	Hochstrasser *et al.* (2001)	DSM-IV and MADRS	11 to 25	Citalopram = 132 (20 mg: 53, 40 mg: 66, 60 mg: 13), placebo= 132	MADRS total score ⩾ 22, confirmed after 3–7 days	48 to 77	Citalopram
Keller *et al.* (1998)^a^	Keller *et al.* (1998)^a^	DSM-III-R and HAM-D	28	Sertraline = 77, placebo = 84	DSM-III-R criteria for MDD for at least 3 weeks, CGI severity score ⩾ 4, CGI improvement score ⩾ 3, and an increase in HAM-D to ⩾4 points higher than the maintenance phase baseline, confirmed within 1 week and by senior clinician	76	Sertraline
Stewart *et al.* (1998)	Stewart *et al.* (1998), McGrath *et al.* (2000), Joliat *et al.* (2004)	DSM-III-R and modified HAM-D	12/26/50	Fluoxetine hydrochloride = 96 (until week 26), 99 (until week 50), 102 (until week 62), placebo = 95 (starting week 12)	Either a modified HAM-D score ⩾ 14 for 3 consecutive weeks or having met the DSM-III-R criteria for MDD for 2 consecutive weeks	50	Fluoxetine
Keller *et al.* (2007) PREVENT study	Kornstein *et al.* (2014)	DSM-IV and HAM-D	34	First 12 months: venlafaxine = 129, placebo = 129; second 12 months: venlafaxine = 32, placebo = 40	HAM-D-17 score > 12 and an HAM-D-17 reduction from acute-phase baseline that was not more than 50% at two consecutive visits or at the last valid visit prior to patient discontinuation	52 to 104	Venlafaxine
Perahia *et al.* (2006)	Fava *et al.* (2009)	DSM-IV, HAM-D-17 and CGI-S	12	Duloxetine = 136, placebo = 142	Increase in the CGI-S score of ⩾2 points compared with randomization and meeting the MINI depression module criteria for major depressive episode at two consecutive visits at least 2 weeks apart	26	Duloxetine
Thase *et al.* (2001)	Nierenberg *et al.* (2004)	DSM-IV and HAM-D-17	8 to 12	Mirtazapine = 76, placebo = 80	Clinician's decision	40	Mirtazapine
Rouillon *et al.* (2000)	Rouillon *et al.* (2000)	DSM-III-R and HAM-D	25	Milnaciprin = 104, placebo = 110	MDD according to DSM-III-R criterion and HAM-D ⩾ 18	52	Milnacipran
Schmidt *et al.* (2000)	Joliat *et al.* (2004)	DSM-IV confirmed with SCID-P, HAM-D-17 and CGI-S	13	Fluoxetine enteric- coated = 190, fluoxetine = 189, placebo = 122	SCID-P major depressive episode module, except for symptom duration and an increase in the CGI-S score ⩾ 2 or relative to the rating before randomization for two consecutive visits	25	Fluoxetine

DSM, Diagnostic and Statistical Manual of Mental Disorders; CGI, Clinical Global Impression scale; MADRS, Montgomery–Åsberg Depression Rating Scale; HAM-D, Hamilton Depression Rating Scale; MDD, major depressive disorder; PREVENT, Prevention of Recurrent Episodes of Depression With Venlafaxine for Two Years; CGI-S, Clinical Global Impression scale – severity; MINI, Mini-International Neuropsychiatric Interview; SCID-P, Structured Clinical Interview for DSM-IV, patient edition.

a Only patients with chronic or double depression were included.

**Table 2.** Demographics, disease course variables, depression subtypes and co-morbidity

	McGrath *et al.* (2000)	Trinh *et al.* (2011)	Hochstrasser *et al.* (2001)	McGrath *et al.* (2006)	Kornstein *et al.* (2014)	Fava *et al.* (2009)	Keller *et al.* (1998)	Stewart *et al.* (1998)	Yang *et al.* (2009)	Nierenberg *et al.* (2004)	Iovieno *et al.* (2011)	Rouillon *et al.* (2000)	Joliat *et al.* (2004)
Demographics													
Age			n.s.	n.s.		n.s.							
Gender	n.s.		n.s.	n.s.	n.s.	n.s.							
Race and ethnicity		Sig											
Disease course variables													
Chronicity and age of onset	n.s.		n.s.	n.s.									
Severity at onset			n.s.				n.s.						
Number of prior episodes			n.s.	n.s.		n.s.	n.s.						
Response pattern	n.s.							Sig		Sig			
Residual symptoms						n.s.	n.s.				n.s.	n.s.	
Specific residual symptoms:													
Phobic anxiety									n.s.				
Depression subtypes													
Typical *v.* atypical depression	Sig^a^			n.s.									
Melancholic subtype	Sig			n.s.									
Double depression							n.s.						
Bipolar II	n.s.												
Co-morbidity													
Anxiety						n.s.							Sig
Somatic pain						Sig							

n.s., No significant interaction term of treatment and predictor or significant subgroup comparisons were reported; Sig, either significant interaction term of treatment and predictor or significant subgroup comparisons were reported.

a Three-way interaction of treatment, neurovegetative symptoms and response pattern was significant.

**Table 3.** Further predictors only investigated once

Predictor	Study	Interaction with treatment
Menopausal status	Kornstein *et al.* (2014)	n.s.
Treatment by general practitioner	Hochstrasser *et al.* (2001)	n.s.
Citalopram dose and concomitant treatment	Hochstrasser *et al.* (2001)	n.s.

n.s., Non-significant.

### Demographics

#### Age and gender

The interaction of both age and gender with treatment (switch to placebo *v.* continuing ADM) failed to reach significance in three studies (Hochstrasser *et al.* 2001; McGrath *et al.* 2006; Fava *et al.* 2009). Gender × treatment interactions failed to reach significance in two further studies (McGrath *et al.* 2000; Kornstein *et al.* 2014). The latter re-examined data from the Prevention of Recurrent Episodes of Depression With Venlafaxine for Two Years (PREVENT) trial dataset where patients were randomized between placebo and venlafaxine maintenance after either 6 or 18 months. The interaction of gender with treatment failed to reach significance at either randomization point.

#### Race and ethnicity

One study investigated the effect of race and ethnicity (Trinh *et al.* 2011). Race and ethnicity had no effect on time to relapse in either continuation and discontinuation arms examined separately. Of note, although the discontinuation effect was significant in the Caucasian group (*p* = 0.001), there was no discontinuation effect in the minority groups. However, power in these groups was low (214 Caucasian patients *v.* 22, 13 and six African American, Latino American and Asian Americans, respectively).

### Disease course

#### Chronicity and age of onset

None of the four studies examining chronicity and age of onset found a significant interaction with treatment (McGrath *et al.* 2000, 2006; Hochstrasser *et al.* 2001; Fava *et al.* 2009). Of note, these studies explored different definitions of chronicity, e.g. a last episode length >2 years (McGrath *et al.* 2000) or having very rare or no remission (McGrath *et al.* 2006).

#### Number of prior episodes

Four studies have investigated the interaction of number of prior episodes and treatment. No significant results were reported (Keller *et al.* 1998; McGrath *et al.* 2000; Hochstrasser *et al.* 2001; Fava *et al.* 2009).

Of note, in the study by Keller *et al.* (1998) the risk of re-emergence of depression during placebo treatment among patients experiencing their first episode was lower (40%) than in patients with recurrent disorder (55%). By contrast, sertraline was equally effective for patients in these subgroups (29% *v.* 24% symptom re-emergence). Although the drug–placebo difference in prophylactic efficacy was higher for patients with recurrent depression (i.e. 31% *v.* 11%), the treatment × prior episode events interaction analysis did not significantly predict time to re-emergence (*p* = 0.25), possibly due to power issues (see also the discussion of meta-analytic results in the Discussion).

#### Severity at onset and episode length

Only two studies report examining an interaction of severity at onset with treatment, both failing to reach significance (Keller *et al.* 1998; Hochstrasser *et al.* 2001). One study found no interaction with episode length (Fava *et al.* 2009).

#### True drug response *v.* placebo response pattern

A pattern of delayed but sustained improvement after antidepressant treatment initiation may distinguish improvements driven by a truly pharmacological effect from those driven by a placebo effect (Quitkin *et al.* 1987). Three studies have examined whether this might in turn predict differential relapse risk after discontinuation. Two studies found consistent subgroup effects, whereby discontinuation increased relapse rates in the likely true drug responders [Nierenberg *et al.* 2004: log-rank = 8.55, degrees of freedom (df) = 1, *p* = 0.003; Stewart *et al.* 1998: log-rank = 22.37; *p* < 0.001 when discontinuing after 12 *v.* 26 weeks; log-rank test score = 8.23; df = 1; *p* < 0.005 when discontinuing after 26 *v.* 50 weeks]. The difference between placebo and continuation groups in patients with initial placebo response patterns was not significant. Nierenberg *et al.* (2004) also found that the relapse risk after discontinuation was larger in patients with true than placebo response patterns (log-rank = 4.87, df = 1, *p* = 0.027). Though these results are suggestive, neither study explicitly tested for interactions. McGrath *et al.* (2006) failed to find a significant interaction between response pattern and discontinuation. McGrath *et al.* (2000) extended the Stewart *et al.* (1998) findings (see below).

#### Residual symptoms at randomization

Residual symptoms, i.e. subthreshold symptoms present at the time of randomization, failed to interact with treatment in four studies (Keller *et al.* 1998; Rouillon *et al.* 2000; McGrath *et al.* 2006; Fava *et al.* 2009) though Rouillon *et al.* (2000) report a trend (*p* = 0.06) after milnacipram discontinuation. Analyses in a further study investigating specific residual symptoms, namely phobic anxiety, also failed to yield significant results (Yang *et al.* 2009).

The lack of interaction with treatment holds for both true residual symptoms (present both at treatment initiation and randomization) and symptoms emerging with treatment (not present at treatment initiation; Iovieno *et al.* 2011).

### Depression subtypes

#### Typical *v.* atypical depression

Neurovegetative symptoms moderated an effect of treatment response specificity (McGrath *et al.* 2000; see also below for response specificity). Amongst patients with typical vegetative symptoms who appeared to have responded specifically to the treatment (a slow, sustained response after treatment onset), there was a large effect of discontinuation (drug–placebo difference; log-rank = 38.8, df = 3, *p* = 1.9 × 10^−8^). This effect was absent in patients with a non-specific response to treatment and in those with atypical vegetative symptoms. The interaction of treatment and neurovegetative symptoms alone was not reported by McGrath *et al.* (2000) and was not significant in a later trial by the same authors (McGrath *et al.* 2006).

#### Melancholic subtype

Two studies investigated the interaction between melancholic depression subtype and treatment. In McGrath *et al.* (2000), patients with melancholic MDD (Diagnostic and Statistical Manual of Mental Disorders, Third Edition Revised; DSM-III-R) appeared to show a larger drug–placebo difference than those with non-melancholic MDD due to increased survival in the melancholic group assigned to maintenance fluoxetine (log rank = 29.3, df = 3, *p* = 1.9 × 10^−6^). However, no interaction was reported and the effect did not survive when controlling for neurovegetative pattern (typical *v.* atypical), even though melancholic and neurovegetative patterns were uncorrelated. Melancholic subtype did not interact significantly with treatment in the study by McGrath *et al.* (2006).

#### Double depression and bipolar II

Neither presence of dysthymia nor a past history of hypomanic symptoms interacted with treatment (Keller *et al.* 1998; McGrath *et al.* 2000). However, Keller *et al.* (1998) only included patients with chronic or double depression and no studies exist that have compared dysthymia and recurrent depression with recurrent depression alone.

### Co-morbidity

Two studies examined the effect of anxiety. Joliat *et al.* (2004) investigated drug- *v.* placebo-treated groups separately. High anxiety increased relapse risk in the discontinuation group (risk ratio = 1.632, *p* = 0.013), but not in the continuation group, though no interaction was reported. Relapse rates were 28.5% *v.* 27.2% and 53.3% *v.* 40.7% for continuation and discontinuation groups with high and low anxiety, respectively. Fava *et al.* (2009) found the interaction of treatment and Hamilton Rating Scale for Depression anxiety/somatization subscore as categorical variable not to be significant.

Somatic pain ratings were found to interact significant with treatment in one study on duloxetine (Fava *et al.* 2009; hazard ratios 0.62 *v.* 0.25 in high- *v.* low-pain groups, *p* = 0.048).

### Other variables

Other variables investigated only once and not found to interact with treatment are presented in Table 3.

## Discussion

Many of the factors reported on here appear to have robust properties as predictors of relapse or recurrence risk independent of medication discontinuation: number of previous episodes (Berlanga *et al.* 1999; Kendler *et al.* 2000; Burcusa & Iacono, 2007; Hardeveld *et al.* 2010), residual symptoms (Hardeveld *et al.* 2010; Nierenberg *et al.* 2010) and other factors all robustly increase the risk of relapse. Clearly, individuals with a higher risk have more potential to benefit from maintenance treatment compared with those with little risk. The presence of such factors, combined with the known strong protective effect of ADM against relapse, is therefore a motivating factor for clinicians and patients alike to continue medications.

However, the relevant question for the individual patient really is about the differential impact of medication: will this particular person benefit from continuing treatment? This requires understanding and predicting not just overall relapse risk, but the specific consequences of medication discontinuation, i.e. effects within the separate arms. Although people with a high risk of relapse have more scope to benefit from maintenance treatment, they may still not respond and therefore not benefit. In addition, discontinuation itself is likely to have an effect on relapse, indicated by the increased risk in the early months following discontinuation, which is independent of length of prior treatment (Viguera *et al.* 1998). Hence, there is no necessary overlap between predictors of relapse independent of treatment (relapse risk overall) and predictors of relapse after antidepressant discontinuation, and we therefore focused on predictors of relapse after discontinuation and the interaction of treatment with a putative predictor in predicting relapse.

Strikingly, our systematic review identified only 13 studies examining the latter, based on only nine datasets. This is thus very poorly understood even though data relevant to this question are routinely available in studies examining continuation and maintenance treatment of depression.

Overall, the state of the field is insufficient to draw either positive or negative conclusions. Nevertheless, a few findings are noteworthy. First, guidelines typically recommend continuation treatment for around 4–9 months (Bauer *et al.* 2013) after the first episode, and longer thereafter. The studies examined here had pre-randomization treatment intervals that were mostly shorter. Amongst those with varying pre-randomization durations none examined the impact of treatment duration directly, meaning that there is a lack of evidence speaking to this point. Several meta-analyses (Viguera *et al.* 1998; Geddes *et al.* 2003; Kaymaz *et al.* 2008; Glue *et al.* 2010; Andrews *et al.* 2011) have, however, examined it, with none finding a significant effect. A newer meta-analysis found an exponential decrease in relapse risk with increasing treatment length (Baldessarini *et al.* 2015). However, this study did not compare discontinuation with maintenance and hence it is unclear how this relates to the discontinuation itself. Furthermore, it is unclear whether the important variable is the time in remission or the time on medication. These are likely to be particularly highly correlated in the studies with shorter initial stabilization which Baldessarini *et al.* (2015) found to have a higher relapse rate. Furthermore, a meta-analysis by the same group (Sim *et al.* 2015) found an increase in the drug–placebo difference with longer pre-randomization stabilization time, a result pointing in the opposite direction. Until more consistent results are available, discontinuation choices should not be determined by considerations of treatment duration alone.

Second, the reviewed studies individually failed to show clear effects of prior episode number. Two meta-analyses have addressed this, with discrepant results. While Viguera *et al.* (1998) found that patients with more prior episodes benefited more from antidepressant treatment, Kaymaz *et al.* (2008) came to the diametrically opposed conclusion, finding that an increasing number of prior episodes instead reduced the prophylactic effect of antidepressants. Using a meta-regression approach, they later found that the odds ratio for relapse after discontinuation compared with continuation in first-episode patients was 0.12, while it was 0.31 in patients in their second or more episodes. Furthermore, Sim *et al.* (2015) found no effect of the estimated number of prior episodes on the drug–placebo effect in continuation and long-term studies. Hence, for patients with multiple past episodes, the situation is unclear: individual studies do not provide a clear picture; and meta-analyses raise the possibility of increased and decreased benefit or no effect at all.

Third, the studies have also not provided clear support for the influence of residual symptoms. This is again surprising. Residual symptom load affects relapse risk overall (Nierenberg *et al.* 2010) and probably accounts for the impact of different definitions of remission on relapse risk (Dunlop *et al.* 2012). Given that patients with high relapse risk should stand more to gain from medication; and given the strong impact on relapse (Geddes *et al.* 2003), one would have expected substantial effects here. It is not inconceivable that placebo treatment response might contribute to this lack of anticipated effects and this would bring attempts to clearly define active treatment response into renewed consideration (Stewart *et al.* 1998; McGrath *et al.* 2000; Nierenberg *et al.* 2004).

The studies by Stewart *et al.* (1998) and Nierenberg *et al.* (2004) both showed that patients with a ‘true-drug response’ with maintained but somewhat delayed improvement profited from active treatment compared with placebo, whereas this difference was not evident in patients with a ‘placebo response pattern’ with very fast initial response that was poorly maintained. While others have argued for a rapid response overall (Szegedi *et al.* 2009), this raises the question of whether aspects of the initial treatment response might differentiate those subjects who do and do not relapse after discontinuation; and this in turn might provide a particularly powerful handle on the identification of true drug response.

Methodologically, to establish the effect of risk reduction after discontinuation of a certain predictor and whether a subgroup identified by the predictor would not benefit from continuous ADM treatment, one would ideally first compute the significance level of the interaction of treatment and predictor and, only if significance is reached, do *post-hoc* comparisons between subgroups correcting for multiple comparisons to identify subgroup difference that gave rise to the significant interaction term. Few studies followed this approach, typically either not reporting the interaction term, or, if the interaction term is reported to be significant, not doing sufficient *post-hoc* comparisons to establish what gave rise to the significant interaction term. Furthermore, corrections for multiple comparisons were not consistently reported.

One possibility for the lack of significant findings is that antidepressant discontinuation has a far stronger effect on relapse rates than any other variable, and that only very large studies or meta/mega-analyses could identify the smaller moderating factors. The strength of the effects for instance of number of prior episodes reported in two meta-analyses (Viguera *et al.* 1998; Kaymaz *et al.* 2008) would seem to speak against this, but the fact that the sign of their finding is in the opposite direction might support the contention. In this vein, there are also experimental reasons for small effect sizes. For instance, antidepressant–placebo response differences have decreased over the past few decades, in part due to recruitment of less severely ill patients into trials (Khan *et al.* 2010). Less ill patients are known to have a smaller relapse risk overall (e.g. Nierenberg *et al.* 2010), and hence are overall less likely *a priori* to benefit from continuation treatment. If treatments are encapsulated, blinding may be partially broken if capsules are intentionally or unintentionally opened and the true drug/placebo identified. This may artificially increase drug–placebo relapse rates and thereby reduce the impact of moderators. Andrews *et al.* (2011) identified two further aspects that may moderate the influence of risk factors. First, they found that relapse risk was higher after discontinuing antidepressants with larger effects on the serotonergic or noradrenergic system. Since most studies in this review used selective serotonin reuptake inhibitors (SSRIs) or serotonin and norepinephrine reuptake inhibitors (SNRIs), the effect on the serotonergic system was likely to be high, potentially decreasing the influence of moderating effects. Second, they suggested the development of oppositional tolerance in response to prolonged drug treatment such as the reduction of intrinsic serotonin synthesis due to antidepressant-induced increases in availability. This could contribute to increased relapse risks after prolonged treatment and thereby counterbalance protective effects of longer treatment.

The present systematic review has some limitations. First, since we only used one database for our search and the database had not access to the full text of all relevant studies for the search, it is possible that we missed eligible studies. There may also be a positive bias due to reporting biases, as positive findings were more likely to be cited in the reviews of which we examined, and as they are more likely to form part of the title of the paper which we based our initial search on. We did not formally evaluate the quality of the included studies as the results were overall weak. A second drawback of the review is the fact that we excluded studies that investigated the effect of psychotherapy on relapse risk after antidepressant discontinuation as this would have confounded the findings given that psychotherapy is known to be effective in preventing relapses (e.g. Hollon *et al.* 2005). In this vein, however, we would like to emphasize that we consider it highly important that patients are informed about alternative treatments, particularly psychotherapeutic ones. Although, as mentioned previously, the presented results might be subject to a positive bias in respect to the reported literature and results of data analyses, the presented view might be overly negative concerning the underlying effect. This probably results from the small sample sizes of the studies mentioned, making the discovery of significant results less probable. Since, in total, sufficient data have been collected, rather than conducting a further medium-sized trial, we recommend the reanalysis of concatenated raw data from previous trials using a machine learning approach, similar to, for example, Chekroud *et al.* (2016). In fact, we are planning to conduct such a mega-analysis, i.e. reanalysing the raw data of a range of identified datasets to investigate relapse predictors after antidepressant discontinuation.

## Conclusion

Maintenance treatment after a remission from depression, particularly after multiple episodes, is the standard of care. However, it is not a panacea. Patients discontinue for a variety of reasons including side effects; and there are indications that tachyphylaxis (Rothschild *et al.* 2009) and even oppositional tolerance may occur (Andrews *et al.* 2011; El-Mallakh & Briscoe, 2012). Individual patients must be provided with good information about the likely course of their own disease trajectory to allow them and their physicians to make informed choices. As we have seen here, few of the predictors of overall relapse risk, surprisingly, appear to differentially predict relapses after continuation *v.* discontinuation. On the one hand, this is clearly due to the scarcity of studies that have attended to the problem. On the other hand, the strong effects on relapse rates of both antidepressants and predictors would have rendered strong interaction effects a distinct possibility. It is hence critical to revisit the existing datasets to re-examine this problem. In doing so, the field can now avail itself of novel techniques both from machine learning and computational psychiatry (Huys *et al.* 2011; Montague *et al.* 2012; Wolfers *et al.* 2015; Chekroud *et al.* 2016) to hopefully provide individually valid predictors of differential risk.

Finally, the list of predictors evaluated so far includes no neurobiological assessments. This should be addressed as such measurements hold great promise in predicting individual outcomes. Farb *et al.* (2011), for instance, found that a simple functional magnetic resonance imaging (fMRI) measure coupled with an emotion manipulation (sad movies) could near perfectly predict relapse overall independently of treatment, and Lythe *et al.* (2015) found that self-blame-related fMRI connectivity features had predictive validity. Other neurobiological features might relate to specific treatments. Clearly, advances in neuroimaging and the neurosciences more generally should be brought to bear on this pressing clinical issue.
